# Targeting Protein–Protein Interactions (PPIs) to Drive Functional Annotation: An Integrative Methodology to Study Senescence-Associated PPIs Using TMPRSS11a as Model Interactor

**DOI:** 10.3390/biom16050714

**Published:** 2026-05-13

**Authors:** Roberto Rosales-Rojas, Christian Fernández, Mariela González-Avendaño, Ariela Vergara-Jaque, Mónica Cáceres

**Affiliations:** 1 Millennium Institute on Immunology and Immunotherapy, Instituto de Ciencias Biomédicas (ICBM), Facultad de Medicina, Universidad de Chile, Independencia 1027, Santiago 8380453, Chile; roberto.rosales@utalca.cl (R.R.-R.); christian.fernandez@ug.uchile.cl (C.F.); 2 Center for Bioinformatics, Simulation and Modeling, Faculty of Engineering, Universidad de Talca, Talca 3460000, Chile; mariela.gonzalez@utalca.cl; 3 Instituo de Ciencias Biomedicas (ICBM), Faculty of Medicine, Universidad de Chile, Santiago 8380453, Chile

**Keywords:** senescence, protein–protein interactions, selective proteomics, nucleotide-binding domain

## Abstract

Protein–protein interactions (PPIs) play a central role in regulating cellular processes. However, the identification and characterization of senescence-associated PPIs remain challenging. In this study, we developed and evaluated an integrative methodology based on selective proteomics to identify PPIs associated with cellular senescence induced by TMPRSS11a. We started from isolated proteins that co-immunoprecipitated with TMPRSS11a and were subsequently identified by mass spectrometry. Building on this dataset, we implemented a workflow combining selective proteomics, structural bioinformatics, and experimental validation. Using this approach, we investigated the interaction between the transmembrane serine protease TMPRSS11a and the chaperone HSPA8. Structural bioinformatics analyses were performed to identify potential residues involved in the interaction interface, and the proximity of the TMPRSS11a–HSPA8 complex was evaluated using an in vitro proximity ligation assay. Our results provide evidence for an interaction between TMPRSS11a and HSPA8 and suggest its association with an enhanced senescence response. Overall, this study presents a workflow to investigate senescence-associated PPIs from proteomics-derived candidate proteins.

## 1. Introduction

Selective proteomics is a strategy for the targeted analysis of specific proteins within complex biological samples [1,2,3,4]. This approach focuses on the thorough study of a subset of proteins, including their structures, functions, and established interactions. Proteins contribute to all kinds of cellular and molecular functions. Their functional diversity is mainly due to their defined structures and unique interaction partners [5,6]. The interactions that occur between proteins can exert absolute control over signaling pathways, allowing for proper physiological function [7,8,9]. Therefore, understanding the nature of protein–protein interactions (PPI) is essential for identifying new molecular targets that can be modulated through pharmacological therapies based on the rational design of small chemical molecules or short peptides that modulate specific contact regions between proteins [10,11].

Aging is a gradual biological process characterized by a progressive decline in the physiological integrity of the organism, which directly impacts the proper functioning of physiological processes. At the molecular level, PPIs, as key components of cellular interaction networks, play a fundamental role in coordinating complex biological processes [12]. To provide a comprehensive study of aging, characteristic hallmarks of its progression have been proposed, including proteostasis and cellular senescence [13]. Proteostasis is a complex molecular mechanism that relies on an extensive network of chaperone proteins and proteolytic systems to maintain proteome homeostasis. Like all biological functions, structural maintenance of proteins also loses efficiency during aging, leading to the accumulation of misfolded proteins and protein aggregates, which promote significant stress for the endoplasmic reticulum (ER) and cells in general. On the other hand, senescence is a cellular mechanism induced by various stress pathways, with common triggers including DNA damage responses (DDR), telomere shortening with each cell replication, oncogene activation, and oxidative stress in cellular compartments [14,15]. In this context, senescent cells acquire a series of distinctive biological features, such as an essentially irreversible cell cycle arrest, increased resistance to apoptosis, metabolic changes, and the release of a senescence-associated secretory phenotype (SASP) [16,17,18]. These cells are present throughout the entire lifespan, from embryogenesis to death [19]; however, during aging, senescent cells accumulate within the organism, which has been studied as the root cause of all age-related diseases [20,21].

Recently, our laboratory identified TMPRSS11a [22] (also known as ECRG1, HATL1), a type II serine protease with a trypsin-like domain, as a novel tissue-specific regulator of cell migration and wound repair. Interestingly, TMPRSS11a expression increases during aging, and its overexpression induces senescence [22]. Moreover, using 22-month-old aged mice, we determined that attenuating expression of the protease improves the repair process and significantly reduces DNA damage foci [22]. Despite these investigations into the function of TMPRSS11a, together with others findings in the literature [23,24,25], the molecular mechanisms by which TMPRSS11a induces senescence remain unknown.

To evaluate the molecular mechanisms by which TMPRSS11a induces senescence, we focused on its potential protein–protein interactions. In this work, we developed an approach based on an integrative and sequential workflow that combines selective proteomics, structural bioinformatics, and targeted experimental validation. This strategy enables the systematic prioritization and characterization of senescence-associated interactors of TMPRSS11A, using a selective proteomics protocol to identify candidate protein–protein interactions (PPIs) and to study these interactions through both in silico and in vitro assays. This protocol was based on 305 proteins identified through co-immunoprecipitation (Co-IP) coupled with mass spectrometry [22]. These proteins were intersected with six databases related to aging and senescence, and we subsequently performed an expression analysis under different senescence stimuli to selectively filter the proteins. Based on our results from the protein intersections and differential expression analyses, we determined that the chaperone HSPA8 [26] was an interesting candidate for further studying its protein–protein interaction with TMPRSS11a. This chaperone is a member of the highly conserved HSP70 family throughout evolution [26,27]. Its subcellular localization has been canonically reported in the cytoplasm, nucleus, and endoplasmic reticulum [26,27]. Structurally, HSPA8 comprises two main domains: the nucleotide-binding domain (NBD), which contains an ATP hydrolysis site, and the substrate-binding domain (SBD), where it can establish transient interactions to assist client proteins in their proper structural folding [26]. In terms of its function, HSPA8 plays a central role in proteostasis, aiding in the structural maintenance of proteins, facilitating the correct structural folding of proteins, and refolding misfolded proteins or peptides resulting from amyloid disaggregation [26,27,28].

## 2. Methodology

### 2.1. Refine Candidates for Protein–Protein Interactions

The 305 proteins co-immunoprecipitated (Co-IP) with TMPRSS11a were previously obtained in our laboratory [22]. All proteins identified by Co-IP coupled to mass spectrometry were initially analyzed using STRING DB [29] to evaluate the predicted PPI associations within the dataset. Subsequently, the molecular functions of all co-immunoprecipitated proteins were determined with the CLUEGO software v2.5.9 [30], which is integrated into Cytoscape v3.9.0 [31]. Using the ‘Positive Regulation of Cellular Senescence’ node as the focal point, a k-nearest neighbor analysis was conducted to identify the cellular and molecular functions that regulate senescence induction through TMPRSS11a overexpression.

A Functional enrichment analysis was performed using the PANTHER v19 (Protein Analysis Through Evolutionary Relationships) Classification System [32,33]. The dataset also corresponds to all proteins identified by Co-IP, which were analyzed against the reference background consisting of all human genes available in the PANTHER database. Enrichment analysis was evaluated using the Gene Ontology (GO) annotation dataset for biological processes [34], based on the GO web resource. Statistical significance of the enrichment was assessed using Fisher’s exact test, and multiple testing correction was applied using the False Discovery Rate (FDR) method [32,35]. Enriched terms were considered significant according to the adjusted *p*-value threshold defined after FDR correction. For visualization purposes, enrichment significance was represented as −log10(FDR), and the top 15 enriched GO biological process terms were plotted. Additionally, a GO term related to the induction of cellular senescence was included in the visualization.

Protein networks were built to find candidates for protein–protein interactions with TMPRSS11a. Additionally, overrepresentation data related to senescence were analyzed across six databases, including SeneQuest ICSA [17], STRING [29], CUEGO [30], Gene Ontology [34], HAGR [36], and OpenTargets [37]. Each dataset from the six databases was cross-referenced with the 305 co-immunoprecipitated proteins serving as the reference. From these network intersections, 22 candidate proteins were identified based on their presence in more than three databases. Relevant biological information for these 22 candidate proteins was obtained using the PPI-MASS platform [38]. Finally, using the ATLAS-SASP server [16], we determined that 10 of the 22 candidate proteins were differentially expressed in the SASP under distinct senescence-inducing stimuli and across different cell lines. All data were sourced from public databases and processed using Python 3 with the Pandas v1.1.5, Numpy v1.19.2, and Matplotlib libraries v3.3.4.

### 2.2. Plasmids

Plasmids encoding human TMPRSS11a (MYC-DDK-tagged, #RC221395) were purchased from Origene, Rockville, MD, USA. Plasmids encoding human TMPRSS11a-EGFP were purchased from GenScript, Piscataway, NJ, USA. GFP-HSPA8 was a gift from Christine Mayr (Addgene plasmid #121161;). Empty vectors pcDNA4/TO or EGFP were from Lonza (Bend, OR, USA).

### 2.3. Cell Culture

HEK293 cells (purchased from ATCC (CRL-1573)) were cultured in Dulbecco’s Modified Eagle’s Medium containing 10% fetal bovine serum (FBS) (HyClone, UT, USA) at 37 °C in a 5% CO2 as described in Fernandez et al., 2021 [22].

### 2.4. Immunofluorescence

The 10,000 Transfected HEK293 cells with empty vector (pcDNA 4/TO), TMPRSS11a, HSPA8, TMPRSS11a:HSPA8 were plated on 12 mm coverslip, fixed in fixative solution (4% *w*/*v* formaldehyde (freshly prepared from paraformaldehyde, Sigma-Aldrich, #158127, St Louis, CA, USA)) in PBS, pH 7.4, cells were blocked with blocking solution containing 4% *w*/*v* nonfat dry milk in PBS, 0.1%Triton X100 (Calbiochem, San Diego, CA, USA, #648462) during one hour, and incubated with respective primary antibody with either anti-phospho histone H2A.X (Ser139) (Millipore, Billerica, MA, USA, #05-636) 1/500, or HSPA8 (Novus, Littleton, CO, USA, #NBP2-12880) 1/1000, Calnexin (Santa Cruz, Santa Cruz, CA, USA, #sc-23954) 1/1000, or FLAG Mouse (Sigma-Aldrich, St. Louis, MO, USA, catalog #F1804). Primary antibodies were detected with Alexa-conjugated secondary antibodies (Invitrogen, #A21244, A27039, A21121, A21242, A21137). Hoechst 33258 (Invitrogen, Carlsbad, CA, USA, #H3569) nuclear stain at 200 ng/mL. Images were acquired with an AxioObserver 7 microscope (Carl Zeiss Microscopy GmbH, Jena, Germany) equipped with a motorized stage and ApoTOME system [39].

### 2.5. Detection of Senescence-Associated Beta-Galactosidase (SA-βGal)

The 10,000 HEK293 cells were washed in PBS and fixed with 2% *w*/*v* paraformaldehyde and 0.2% *v*/*v* glutaraldehyde for 5 min. Then, the cells were processed according to Fernandez et al., 2021 [22].

### 2.6. Modeling of Protein–Protein Interaction

To evaluate the physical association between the human TMPRSS11a and human HSPA8, a structural model was built with ColabFold v1.5.5 using AlphaFold2-multimer [40]. A multiple sequence alignment was generated with MMseqs2 [41] and HHsearch [42]. The best model was selected as that with the highest pLDDT AlphaFold2 score, and it was evaluated through the Ramachandran plot. The contact interface complex predicted by AlphaFold2 was corroborated using RaptorX [43] and BIPSI+ [44]. The structural model generated for TMPRSS11a was compared with the previous model of the catalytic domain reported [22], exhibiting an RMSD lower than 1 Å. The structural model predicted for HSPA8 was compared with the PDB structure ID: 5FPN, exhibiting an RMSD less than 1.5 Å. To refine the protein–protein docking, 5000 models were generated through a Monte Carlo-based multi-scale docking algorithm implemented in Rosetta [45,46]. The best 100 models ranked based on the Rosetta score I_sc were clustered using the VMD plugin iTrajComp v1.0 with a cutoff value of 5 Å. The centroid of the most populated cluster was selected as the final structure for the complex TMPRSS11a:HSPA8.

### 2.7. Molecular Dynamics Simulation

The conformation selected by molecular docking was used as the starting structure to perform three independent molecular dynamics (MD) simulations. The complex was solvated with explicit TIP3P water molecules and ionized by adding 0.15 M of NaCl (sodium and chloride ions) to the aqueous phase to ensure charge neutrality. The initial configuration of the system was optimized by means of 30,000 steps of energy minimization, followed by an equilibration and production simulation using an isobaric-isothermal ensemble. Soft harmonic restraints were applied to the complex (backbone and sidechains) during the first 8 ns of the simulation, which were gradually decreased from 20 to 0 kcal mol^−1^ Å^−2^. The distance between interface contacts for the complex TMPRSS11a:HSPA8 was also restrained using the collective variable module (Colvars) [47] during the first 150 ns of the simulation. The colvars force decreased from 50, 0.5, and 0 kcal mol^−1^ Å^−2^, using three windows of 50 ns for each stage. Finally, three replicas of production simulations were performed without restraints to evaluate the free behavior of the complex, totaling 658 ns of simulation. All MD simulations were performed using NAMD v2.14 [48], applying the all-atom CHARMM36 force field [49]. The Langevin thermostat and barostat were used to maintain a constant temperature and pressure at 300 K and 101.325 kPa (1 atm), respectively [50]. Electrostatic interactions were set to 8–9 Å, applying the particle-mesh Ewald method and hydrogen covalent rigid bond restrictions [51]. The equations of motion were integrated over 4 fs using the repartitioning mass method [52]. The system was structurally analyzed using VMD v1.93 through tcl/Tk [53] and PyMOL v1.8.4 (Schrödinger, LLC, New York, NY, USA) through Python3. The most prevalent residue pairs interactions established between TMPRSS11a and HSPA8 were determined with the get_contacts v1.0 plugin implemented in Python v3.6.13.

### 2.8. Proximity Ligand Assay

The assays were performed using the same protocol with 10,000 transfected HEK293 cells, and the Duolink in Situ Red Starter Kit Mouse/Rabbit (Sigma-Aldrich catalog #DUO92101). Cells were fixed for 10 min with 4% *w*/*v* formaldehyde (freshly prepared from paraformaldehyde Sigma-Aldrich #158127), then incubated with triton 0.1% for 45 min, followed by incubation for 1 h at 37 °C in blocking solution. All subsequent incubations were performed in a humid chamber. Mouse anti-HSPA8 (Novus, catalog #NBP2-12880) and rabbit anti-FLAG (Sigma-Aldrich, catalog #F7425) primary antibodies were added, and samples were incubated for 3 h at room temperature following the manufacturer’s instructions. After two washes for 5 min each, the samples were incubated with secondary antibodies coupled with oligonucleotides for 1 h at 37 °C. After two washes, the ligation solution was added and the samples incubated for 30 min at 37 °C, followed by 100 min incubation with the ligation solution at 37 °C. The samples were mounted, and images were acquired by confocal microscopy. Fluorescence intensity was quantified using the ImageJ/Fiji2 Linux bundled with Java8 software (Madison, WI, USA) and expressed as fluorescence intensity per cell as described. The acquisition settings were identical for all images.

### 2.9. Statistical Analysis

All experiments were independently repeated at least three times. Results are presented as mean ± standard deviation (n = 3). Statistical comparisons between groups were performed using two-way analysis of variance (ANOVA), followed by Tukey’s post hoc test for multiple comparisons. All analyses were conducted using GraphPad Prism software (version 8.0.2; GraphPad Software, San Diego, CA, USA), considering a *p*-value < 0.05 as statistically significant.

## 3. Results

### 3.1. Workflow for Identifying Protein–Protein Interactions Related to Senescence

**Figure 1:**
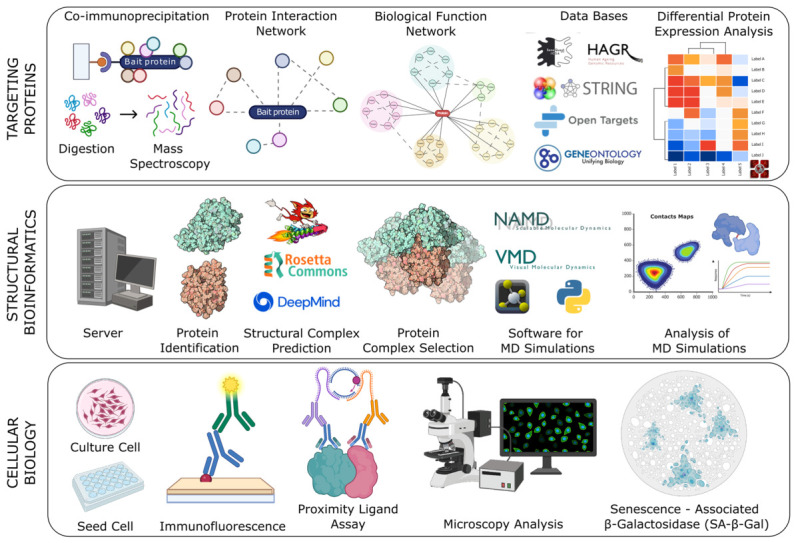
Workflow used to identify Senescence-associated TMPRSS11a protein–protein interaction. Targeting proteins: Details of the main methodologies used to filter candidate proteins for interaction with TMPRSS11a. Structural bioinformatics describes the methodologies and software used to characterize the structural interaction between both proteins. Cellular biology outlines the methodologies for validating the identified interaction and its association with senescence.

To drive functional annotation, we established a selective proteomics-based workflow combining structural bioinformatics and cell biology approaches to characterize interaction interfaces and validate their functional impact on senescence, underlying TMPRSS11a-induced senescence (Figure 1).

### 3.2. Candidates for Protein–Protein Interactions with TMPRSS11a

**Figure 2:**
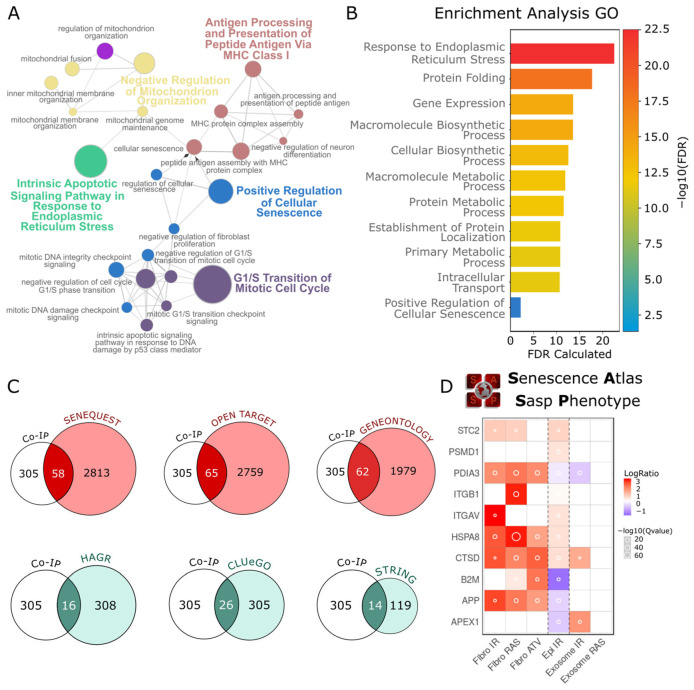
Identification of candidate proteins for interaction with TMPRSS11a. (**A**) Subnetwork of molecular and cellular functions annotated for the 305 proteins obtained through LC-MS/MS coupled with Co-IP, as described in [22]. This subnetwork comprises only the neighborhood of molecular functions centered on “Positive Regulation of Cellular Senescence”. (**B**) GO enrichment analysis of the most overrepresented molecular functions among the 305 candidate proteins. The molecular functions displayed correspond to those with an FDR *p* < 0.05. (**C**) Intersection of protein networks using the 305 proteins co-immunoprecipitated with TMPRSS11a against subsets from the SeneQuest ICSA, GeneOntology, STRING, HARG, OpenTargets, and CLUeGO databases. The intersections show the number of intersected proteins for each case. (**D**) Analysis of protein expression in response to different senescence induction, such as X irradiation, RAS overexpression, and Atazanavir (ATV), using the ATLAS SASP platform. LogRatio indicates the expression level, while −log(QValue) represents measurement reliability.

Using co-IP of TMPRSS11a coupled with LC–MS/MS, as previously reported by our laboratory [22], we obtained 305 proteins as potential candidates for interaction. We determined their cellular and molecular functions using the Gene Ontology and Kyoto Encyclopedia of Genes and Genomes (KEGG) databases. Notably, our analysis revealed nodes corresponding to the Positive Regulation of Cellular Senescence. Using the k-nearest neighbors (KNN) method, we constructed a subnetwork based on the identified nodes, determining four additional biological functions closely associated with senescence induction, such as endoplasmic reticulum stress responses, G1/S cell cycle arrest, negative regulation of mitochondrial organization, and immune system recognition peptides (Figure 2A). Subsequently, Functional enrichment analysis of the 305 candidate interaction proteins was performed using the PANTHER Overrepresentation Test, and enrichment significance was represented as −log10(FDR), and the top 15 enriched GO biological process terms were plotted. Additionally, a GO term related to the induction of cellular senescence was included in the visualization (Figure 2B). Based on the false discovery rate (FDR), we found that the most significant function was associated with endoplasmic reticulum stress responses, protein folding, further highlighting positive regulation of cellular senescence (Figure 2B).

To identify a set of proteins specifically interacting with TMPRSS11a, we used six datasets related to senescence and aging, namely the SeneQuest ICSA [17], GO [34], STRING [29], HAGR [36], OpenTargets [37], and ClueGO [30] databases. Each of these subsets was intersected with the 305 reference proteins (Figure 2C). This approach resulted in the identification of 241 proteins from the original set, of which 172 were unique. Additionally, we found a group of 22 proteins that appeared three or more times across the intersected protein sets. Using the ATLAS SASP platform, we studied the expression of proteins related to SASP, one of the key features of senescence. We found that 10 of them were overexpressed under three different senescence stimuli: X-irradiation (IR), inducible RAS overexpression (RAS), and atazanavir treatment (ATV) (Figure 2D). Based on their frequency across the subsets aging and senescence-related, and their individual expression responses to senescence stimuli, we identified these 10 candidate proteins as potential interaction partners of the protease. Based on its differential upregulation (red-colored squares) under the IR, RAS, and ATV treatments, as well as its statistically significant increase indicated by the circle size in the analysis, the chaperone HSPA8 was selected as a promising candidate to evaluate its interaction with the protease TMPRSS11a and its potential role in the induction of senescence. Based on its differential upregulation (red-colored squares) under the IR, RAS, and ATV treatments, as well as its statistically significant increase indicated by the circle size in the analysis, the chaperone HSPA8 was selected as a promising candidate to evaluate its interaction with the protease TMPRSS11a and its potential role in the induction of senescence. As previously mentioned, the HSPA8 chaperone plays a crucial role in maintaining the structural integrity of the proteome, thus contributing to endoplasmic reticulum stress responses, as revealed by the GO enrichment analysis.

### 3.3. Domains, Motif, and Endoplasmic Reticulum Localization of TMRSS11a, HSPA8, and Their Complex

**Figure 3:**
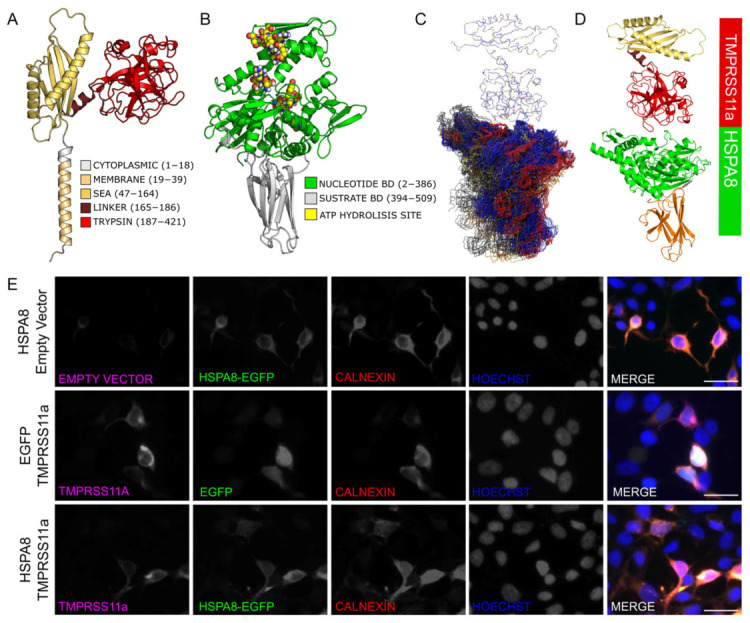
Domain, motif, and RE localization of TMPRSS1a and HSPA8. 3D structures of (**A**) TMPRSS11a and (**B**) HSPA8, depicted as cartoon representations that highlight their distinct functional regions. (**C**) Representative conformations from the molecular docking clustering of TMPRSS11a and HSPA8 based on RMSD. (**D**) Final structural model of the PPI, with the catalytic domain of TMPRSS11a in red and the NBD domain of HSPA8 in green. (**E**) Representative immunofluorescence images of Calnexin (red) in HEK293 cells co-transfected with eGFP-HSPA8 (green) and TMPRSS11a-FLAG (purple). TMPRSS11a was detected using an anti-FLAG antibody. Cells transfected with eGFP alone were used as a control. Cell nuclei were stained with Hoechst (blue), and endogenous Calnexin was used to identify the ER. For all microphotographs, magnification 40× and scale bar = 5 μm. n = 3.

To study the preferred structural conformation between the protease TMPRSS11a and the chaperone HSPA8, we implemented a workflow that included in silico and cellular biology experiments. The three-dimensional structure of the catalytic domain of TMPRSS11a reported in [22] was determined through homology modeling using PDB ID: 2QO5 as a template (Figure 3A). The three-dimensional coordinates of HSPA8 were obtained from PDB structure ID:5FPN (Figure 3B). To generate an initial representative model of the TMPRSS11a:HSPA8 complex, we utilized the AlphaFold2 algorithm. We then refined the initial conformation using Rosetta software v2021.16.61629 through its protein–protein docking protocol. The refined models were clustered into five clusters based on their RMSD values (Figure 3C). According to the Rosetta score and Ramachandran plot, we selected a final conformation to represent the TMPRSS11a:HSPA8 complex (Figure 3D). Our structural analyses indicate that this protein–protein interaction occurs specifically between the catalytic domain of TMPRSS11a and the nucleotide-binding domain (NBD) of HSPA8. suggesting a mechanistic pathway with significant therapeutic potential, revealing key polar interactions that modulate the complex association.

To validate the endoplasmic reticulum (ER) localization of TMPRSS11a and HSPA8, we employed a heterologous model of HEK293 cells co-transfected with eGFP-HSPA8 and FLAG-TMPRSS11a (Figure 3E). Immunofluorescence analyses were performed using Hoechst to visualize nuclei and Calnexin as an ER marker. Quantitative co-localization analysis was conducted using ImageJ software v1.53t by splitting fluorescence channels, followed by Pearson’s correlation coefficient estimation using the JACoP plugin. This analysis revealed a strong positive correlation between TMPRSS11a and Calnexin (r = 0.916 ± 0.037), as well as between HSPA8 and Calnexin (r = 0.948 ± 0.031), indicating a high degree of spatial overlap. In contrast, lower correlation values were obtained for the control EGFP/Calnexin (r = 0.582 ± 0.07), calculated from n = 3 with at least 30 cells per condition. Consistently, both HSPA8 and TMPRSS11a localized to the ER when expressed individually and in co-transfected cells, supporting their presence within the same subcellular compartment.

### 3.4. Protein–Protein Docking and Co-Localization of the TMPRSS11a:HSPA8 Complex

**Figure 4:**
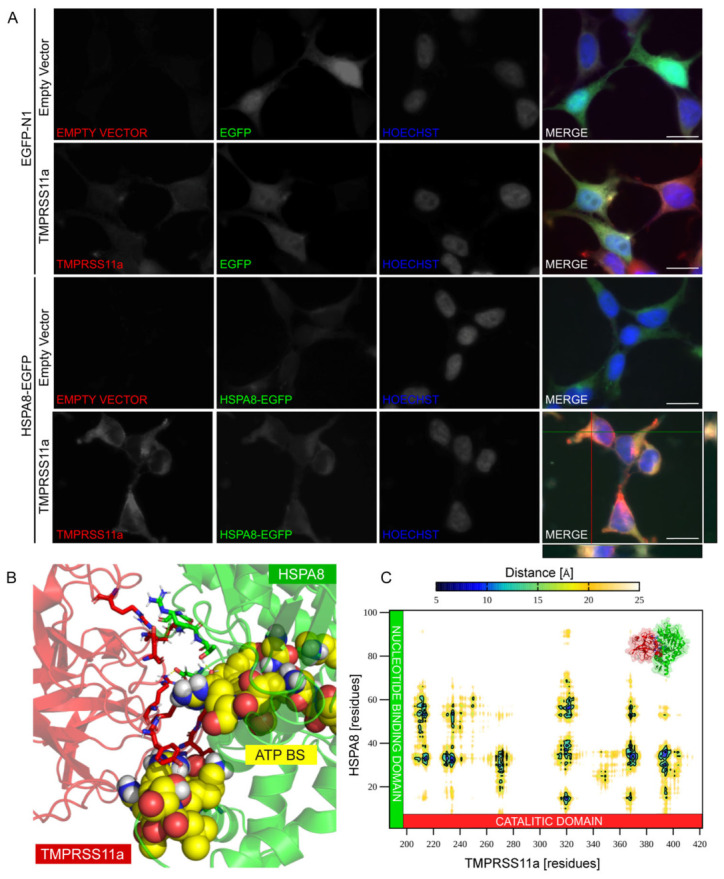
Localization of TMPRSS11a, HSPA8, and contact interface of the TMPRSS11a:HSPA8 complex. Immunofluorescence of the TMPRSS11a, HSPA8 in HEK293 cells co-transfected with eGFP-HSPA8 (green) and FLAG-TMPRSS11a (red). Cell nuclei were stained with Hoechst (blue). (**A**) Immunofluorescence of HEK293 cells transfected with empty vector and EGFP vector as control, transfected with eGFP-HSPA8, transfected with TMPRSS11a and co-transfected with TMPRSS11a and HSPA8. For all microphotographs, magnification 63× and scale bar = 5 μm. n = 3. (**B**) Interphase of the site interaction showing the view of the TMPRSS11a:HSPA8 complex. Red licorice indicates interaction residues from TMPRSS11a, green licorice shows interaction residues from HSPA8, and yellow VDW representation highlights residues corresponding to the ATP-hydrolysis binding site (ATP BS) in the NBD domain of HSPA8. (**C**) 2D contact map displaying the average distance between the centers of mass of residues 200–421 in TMPRSS11a and residues 1–100 in HSPA8 at the interaction site.

To further investigate the co-localization of TMPRSS11a and HSPA8, we employed immunofluorescence and confocal microscopy using HEK293 cells transfected with double empty control which clearly exhibited eGFP expression; cells transfected with FLAG-TMPRSS11a and eGFP; transfection with eGFP-HSPA8 and empty vector pcDNA^TM^4/TO, showing HSPA8 expression without the presence of TMPRSS11a; and finally, the co-transfection of eGFP-HSPA8 and FLAG-TMPRSS11a using anti-FLAG antibody to detect de expression of TMPRSS11a (Figure 4A). Prominent co-localization was observed between TMPRSS11a and HSPA8, supported by a high Pearson’s correlation coefficient r = 0.932 ± 0.034; *p* ≤ 0.001 calculated from n = 3 with at least 30 cells per condition. In contrast, lower correlation values were obtained for the controls (EGFP/pcDNA4/TO r = 0.261 ± 0.06, HSPA8/ pcDNA4/TO r = 0.298 ± 0.051, EGFP/TMPRSS11a r = 0.825 ± 0.041), quantified using ImageJ with the JACoP plugin. Notably, some correlation between EGFP and TMPRSS11a was also observed due to the widespread distribution of the EGFP signal throughout the cell. The nuclei of the cells were stained with Hoechst. Through this experiment, we determined that the specific compartment for the co-localization of TMPRSS11a and HSPA8 is the endoplasmic reticulum. Concerning the physical association of TMPRSS11a and HSPA8, our docking studies reveal a protein–protein interaction between the catalytic domain of TMPRSS11a and the NBD domain (and not within the SBD domain, as anticipated) of HSPA8 (Figure 4B). Utilizing the refined models of the TMPRSS11a:HSPA8 complex generated with Rosetta, we constructed a 2D interaction map that illustrates how the predicted conformations interact through specific residues in both proteins (Figure 4C). Notably, our structural representative model exhibits a blockage at the ATP hydrolysis site in HSPA8 due to its interaction with TMPRSS11a, predominantly involving residues 200–280 and 310–410 of the protease, and residues 15–80 of the chaperone. This interaction suggests a potential steric hindrance affecting ATP hydrolysis by HSPA8, potentially influencing its role in supporting protein folding within the endoplasmic reticulum.

### 3.5. MD Simulation and Proximity Ligand Assay of the TMPRSS11a:HSPA8 Complex

**Figure 5:**
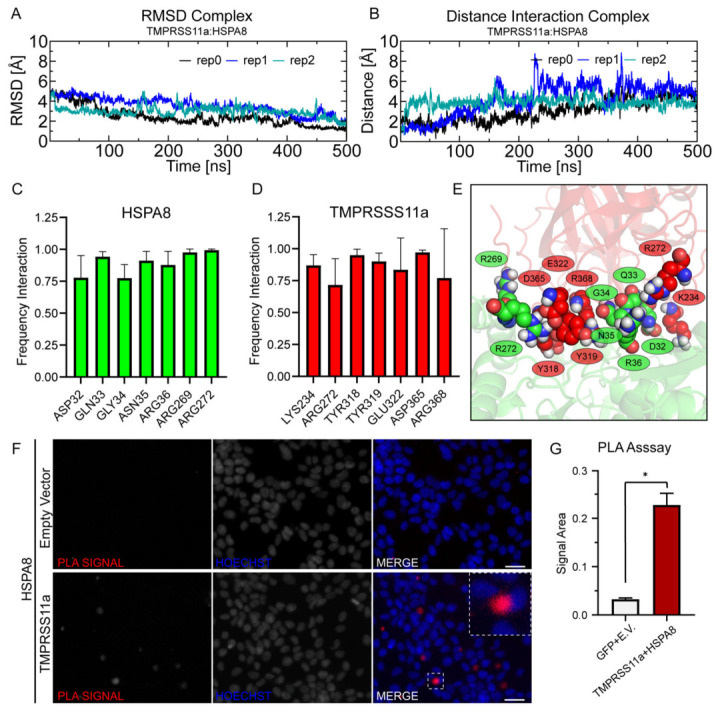
MD simulation and PLA assay of TMPRSS11a:HSPA8 complex. (**A**) RMSD analysis over the simulated time for the three MD replicates. (**B**) Distance calculations between the consensus hot spot of the PPI across the three simulation replicates. (**C**) Frequency of interaction between key HSPA8 residues and TMPRSS11a. (**D**) Frequency of interaction between key TMPRSS11a residues and HSPA8. (**E**) Representative conformation of the protein–protein interaction interface between TMPRSS11a and HSPA8. TMPRSS11a is depicted in red cartoon representation, while HSPA8 is shown in green. Key residues are highlighted using VDW representation. The protein–protein interaction was detected in situ using PLA. This assay utilized the Duolink detection kit with a polyclonal rabbit anti-FLAG antibody for TMPRSS11a and a monoclonal mouse anti-HSPA8 antibody. For all microphotographs, magnification 20× and scale bar = 5 μm. (**F**) Representative images of PLA signal from HEK293 transfected with empty vector as a control and from HEK293 cells co-transfected with TMPRSS11a and HSPA8. (**G**) Graph showing the quantification of the PLA signal area, n = 3. * indicates statistically significant difference.

To identify the predominant interactions within the TMPRSS11a:HSPA8 complex, molecular dynamics simulations were performed using three independent replicas, each demonstrating stable structural conformations. The stability of the complexes was assessed by measuring the RMSD of the backbone for both proteins (Figure 5A). We then analyzed all inter-residue pair distances, applying a 5 Å cutoff to identify non-bonded interactions. Across all replicates, we observed that the protein–protein interaction persisted throughout the 500 ns of MD simulation, with the calculated distances indicating a stable complex interaction (Figure 5B). For the chaperone HSPA8 interacting with TMPRSS11a, we identified the consensus interaction residues: ASP32, GLN33, GLY34, ASN35, ARG36, ARG269, and ARG272 (Figure 5C). In the interaction of TMPRSS11a with HSPA8, the consensus residues identified were LYS234, ARG272, TYR318, TYR319, GLU322, ASP365, and ARG368 (Figure 5D). A representative conformation of the TMPRSS11a:HSPA8 interface region, highlighting the key residues in both proteins, is shown in Figure 5E. Molecular dynamics simulations demonstrated the stability of the complex over time, confirming the interaction site identified in our docking simulations. Additionally, we identified three pairs of residues that maintain the interaction of this molecular complex, as evidenced across the MD replicates: TMPRSS11a:ASP:365-342:ARG:HSPA8, TMPRSS11a:ARG:368-53:ASP:HSPA8, TMPRSS11a:ASP:394-36:ARG:HSPA8. Among the interactions examined, we found that the primary contributors to maintaining the protein–protein interaction are hydrogen bonds and electrostatic interactions. All identified residues in TMPRSS11a are structurally exposed on the front region of the protease’s catalytic domain. Likewise, all identified residues in HSPA8 are located on the front region of its ATP-binding domain.

A PLA assay was performed to confirm the TMPRSS11a-HSPA8 proximity. This assay facilitates the in situ detection of protein associations when two proteins are located within a 30 nm (or less) distance. Our analyses showed no PLA signal under control conditions, while a positive signal was detected when TMPRSS11a and HSPA8 were co-transfected (Figure 5F). Quantification of the PLA signal area revealed statistically significant differences between the control condition and the co-expression of both proteins (Figure 5G). This assay confirms the proximity of TMPRSS11a and HSPA8, demonstrating a PPI between the protease and the chaperone. Our data suggests that the proximity of these proteins in the endoplasmic reticulum may lead to a physical PPI, resulting in proteostasis disruption. This established interaction could contribute to the initiation or maintenance of the senescent phenotype in cells.

### 3.6. Complex TMPRSS11a:HSPA8 Interaction and Its Role in Senescence

**Figure 6:**
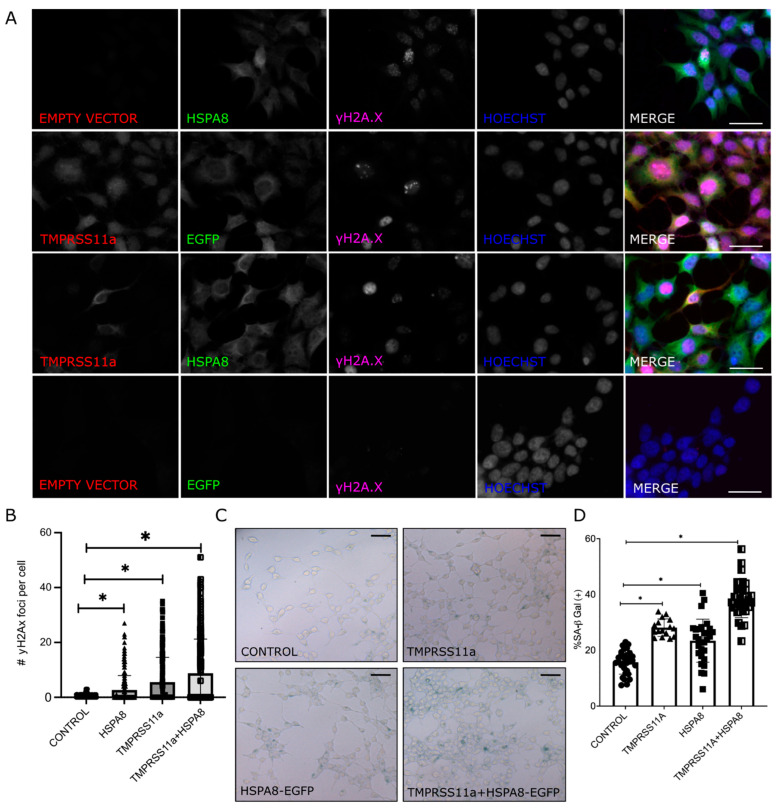
Immunofluorescence of γH2A.X. and SA-βGal activity using the overexpression of TMPRSS11a and HSPA8. HEK293 cells were co-transfected with EGFP-HSPA8 and FLAG-TMPRSS11a. By immunofluorescence, we observed the expression of TMPRSS11a using anti-FLAG (red); and anti γH2A.X (purple) was used as a senescence marker and cell nuclei were stained with Hoechst (blue), (**A**) Immunofluorescence of γH2A.X in HEK293 cells transfected with eGFP-HSPA8, TMPRSS11a or both HSPA8 and TMPRSS11a (**B**) Quantification of γH2A.X foci per cell in HEK293 cells in control cells (transfected with empty vector pcDNA4/TO, HSPA8 transfected cells, TMPRSS11a, transfected cells and co-expressed TMPRSS11 and HSPA8. Both individual expression and co-expression increased the number of γH2A.X foci compared to control, with the highest levels observed in co-expressed cells. Statistical significance is indicated by asterisks. (**C**) Representative images of the SA-βGal assay, including empty vector (pcDNA4/TO) as control, HSPA8, TMPRSS11a, and co-transfection conditions. (**D**) Quantification of percentage of positive cells for SA-βGal activity, showing statistically significant differences between control cells and the overexpression of HSPA8, TMPRSS11, and both proteins. * indicates statistically significant difference.

To investigate the relationship between TMPRSS11a and HSPA8 in cellular senescence, we evaluated DNA damage foci using phosphorylated ser139 H2AX and the activity of the senescence-associated enzyme β-Galactosidase (SA-βGal), both well-used markers for senescence detection. Using HEK293 transfected with TMPRSS11a, HSPA8, or an empty vector as controls, we performed immunofluorescence using anti-γH2A.X to detect DNA damage foci. We observed that cells expressing eGFP-HSPA8, FLAG-TMPRSS11a, or co-expressing both proteins (Figure 6A) showed an increased number of γH2A.X foci per cell, with the highest number of foci observed in co-expressed cells (Figure 6B), suggesting activation of a DNA damage response that could trigger cellular senescence. To further assess senescence, we employed the gold-standard assay for detecting senescent cells, the activity of the senescence-associated enzyme β-Galactosidase (Figure 6B). We quantified the percentage of SA-βGal positive cells and found that cells expressing TMPRSS11a (28.14. ± 1.86) and HSPA8 (23.42 ± 1.56) had a significantly higher proportion of SA-βGal positive cells compared to those transfected with the empty vector (12.44 ± 1.54). Cells co-expressing both TMPRSS11a and HSPA8 had the highest percentage (38.47 ± 1.45). Moreover, this result showed that the co-expression of both proteins amplifies the senescent response in cells, suggesting a synergistic collaboration in the interaction between the protease and the chaperone (Figure 6C). Interestingly, it has been described that both proteins are endogenously overexpressed during aging [22,28].

## 4. Discussion

The advances in bioinformatics and artificial intelligence-based tools have enabled the characterization of an unprecedented number of protein sequences and structures [54]. However, the pace at which these data are generated exceeds the current capacity to interpret them within a biological context, posing significant challenges to elucidating protein functions and their interactions [54,55,56]. Currently, proteomic platforms are essential for functional protein annotation. These tools allow tracking the relationship between proteins and biological functions by integrating information such as expression profiles, identification of PPIs, and function assignment based on sequence and structural features. For protein annotation, examples such as GO [34] or UniProt [57] enable a meta-search of proteomic information using a list of proteins as input. Regarding expression profiling, global platforms such as ATLAS-SASP [16] have been developed to investigate the expression patterns of proteins involved in SASP release. For protein–protein associations, resources like STRING DB [29] or PPI-MASS [38] facilitate the identification of physical or functional interactions derived from a set of candidate proteins against a specific target.

Given that the proteomic information described above is distributed across multiple computational resources, this study developed and validated an innovative methodology that enables the selective integration of proteomic platforms for functional protein annotation. This protocol is grounded in the principle that proteins act cooperatively, participating in signaling cascades mediated by the formation of protein complexes within specific physiological contexts. Within this framework, the proposed methodology establishes the relationship between biological functions and PPI interactions from a set of proteins obtained through Co-IP. This approach enables the identification of protein–protein interactions and provides a robust strategy to infer biologically relevant functions by integrating structural, functional, and experimental data. In this way, the protocol facilitates a more precise interpretation of the molecular mechanisms underlying specific biological processes. One of the limitations of this work is that the Co-IP can generate, in some cases, false positives from nonspecific binding and false negatives due to transient interaction during cell lysis. One of the approaches to minimize this problem is to perform a PLA assay to determine the proximity of less than 40nm. Moreover, in the future, tandem affinity purification (TAP) coupled with mass spectrometry enables systematic identification of protein complexes with reduced background [58]. Likewise, native electrophoresis-based techniques, such as blue native PAGE, allow the separation and characterization of intact protein complexes under non-denaturing conditions. Incorporating such complementary approaches as part of a general methodology can enhance both the coverage and reliability of PPI detection [59].

In this context, TMPRSS11a overexpression has been linked to senescence induction in both in vitro and in vivo models [22]. Proteomic databases such as UniProt [57], STRING [60], and the literature [23,24] further associate TMPRSS11a with cell cycle arrest at the G1 phase. However, the mechanisms by which TMPRSS11a triggers senescence remain largely unknown. To elucidate these mechanisms, we applied our protocol to analyze protein–protein interactions and biological functions specifically annotated for senescence, enabling a focused investigation of the molecular pathways involved. Based on this approach, we identified the chaperone HSPA8 as a potential interactor of the protease TMPRSS11a.

Consistently, experimental evidence showed that the senescent phenotype was enhanced in our SA-βGal assays when both proteins were overexpressed (Figure 6). The PLA signals were expressed under the same conditions (Figure 5). Immunofluorescence analysis revealed the co-localization of both proteins within the endoplasmic reticulum (Figure 3). Structural bioinformatics analysis indicated a steric hindrance at the ATP-binding domain of HSPA8 (Figure 4), suggesting that this interaction may prevent the chaperone from performing its canonical role in protein maintenance and proteostasis. These findings are consistent with our observation of endoplasmic reticulum stress responses, which emerged as the most prominent biological processes among the 305 proteins co-immunoprecipitated with TMPRSS11a in the GO Functional enrichment analysis. Furthermore, this analysis highlighted a strong association with pathways involved in endoplasmic reticulum proteostasis, including protein folding, maturation, and ER-associated degradation, along with broader transcriptional and metabolic programs characteristic of the unfolded protein response [12,13] (Figure 2). Our data indicate that the mechanisms of TMPRSS11a-induced senescence may be linked to a loss of proteostasis, driven by its physical interaction with HSPA8, a key chaperone in maintaining protein homeostasis. It is noteworthy that both TMPRSS11a [22] and HSPA8 [28] are endogenously overexpressed during aging [22,61,62,63]. Interestingly, imbalances in protein homeostasis can lead to a loss of proteostasis, triggering endoplasmic reticulum stress responses that directly impact the onset and maintenance of senescent characteristics [12,13]. In this context, the PPI described in this study suggests alterations in the NBD domain of HSPA8 resulting from its interaction with the catalytic domain of TMPRSS11a, thereby potentially impairing the canonical chaperone function. This may further exacerbate proteostasis imbalance by disrupting protein folding within the endoplasmic reticulum, representing an additional mechanism contributing to cellular stress and reinforcing the senescent phenotype [64,65].

Aging research primarily focuses on the physiological mechanisms driving its progression [12,13]. Notably, two key hallmarks of aging are cellular senescence and the disruption of proteostasis [13]. These two features are interrelated: senescence, a cellular response to various stressors, and the loss of proteostasis, which results in stress within cellular compartments, particularly the endoplasmic reticulum, due to the accumulation or aggregation of misfolded proteins [18,64,65]. To manage this stress, the ER initiates an unfolded protein response (UPR), leading to reduced protein synthesis, ER enlargement, and the export of misfolded proteins [13,18,64]. The UPR involves three main signaling pathways, allowing cells to choose between apoptosis and cellular senescence based on the expression of molecular sensors [66,67]. This mechanism is particularly active during senescence, playing a crucial role in the initiation and maintenance of the senescent phenotype [68,69]. The loss of proteostasis is significantly involved in both aging and cellular senescence [18,66,69,70,71].

## 5. Conclusions

In conclusion, our study introduces an integrative methodology that combines computational proteomics and experimental validation to elucidate functionally relevant protein–protein interactions. By selectively integrating multiple source proteomics platforms, the proposed protocol enables the contextual interpretation of protein interactions and their associated biological functions. Applied to the study of TMPRSS11a, this approach identified its interaction with the chaperone HSPA8, offering mechanistic insight into senescence through the possible disruption of proteostasis; two interconnected hallmarks of aging. Beyond this specific case, our methodology provides a scalable protocol for functional protein annotation and the systematic exploration of molecular mechanisms in complex biological systems.

## Data Availability

The original contributions presented in this study are included in the article. Further inquiries can be directed to the corresponding authors.
